# Comparison of olanzapine-induced weight gain and metabolism abnormalities between topiramate and vitamin C in patients with schizophrenia: a preliminary study

**DOI:** 10.3389/fpsyt.2023.1152953

**Published:** 2023-05-12

**Authors:** Jinling Zhang, Shu Chen, Jia Chen, Handi Zhang, Wen-Wang Rao

**Affiliations:** ^1^Mental Health Center of Shantou University, Shantou, Guangdong, China; ^2^The Fourth People's Hospital of Chengdu, Chengdu, Sichuan, China; ^3^Department of Preventive Medicine, Shantou University Medical College, Shantou, Guangdong, China

**Keywords:** topiramate, vitamin C, olanzapine, weight gain, metabolism abnormalities, schizophrenia

## Abstract

**Background:**

Topiramate (TPM) may reduce olanzapine (OLZ)-related weight gain and metabolism abnormalities in patients with schizophrenia. However, differences in the efficacy of OLZ-related weight gain and metabolism abnormalities between TPM and vitamin C (VC) are not clear. This study aimed to investigate whether TPM is more effective than VC in reducing OLZ-induced weight gain and metabolic abnormalities in patients with schizophrenia and explore their patterns.

**Methods:**

This was a 12-week longitudinal comparison study in OLZ-treated patients with schizophrenia. Twenty-two patients who received OLZ monotherapy plus VC treatment (OLZ + VC group) was matched to 22 patients who received OLZ monotherapy plus TPM treatment (OLZ + TPM group). Body mass index (BMI) and metabolism indicators were measured at baseline and 12-weeks follow-up.

**Results:**

A significant difference in triglyceride (TG) levels at different time points (pre-treatment: *F* = 7.89, *p* = 0.008; 4-weeks treatment: *F* = 13.19, *p* = 0.001; 12-weeks treatment: *F* = 54.48, *p* < 0.001) was found. Latent profile analysis demonstrated that a 2-class model for OLZ + TPM group (high vs. low BMI in the first 4 weeks) and OLZ + VC group (high vs. low), respectively.

**Conclusion:**

Our findings suggested that TPM could better mitigates OLZ-induced increase in TG levels. The trajectories of change also differed in all metabolic indexes over time between the two groups.

## 1. Introduction

Schizophrenia is a serious psychiatric disorder (1, 2), with a lifetime prevalence of approximate 0·6% in China (3, 4), which could significantly impact metabolic changes (5, 6). The Global Burden of Disease study estimated that schizophrenia accounted for about 12.66 million disability-adjusted life years (7). Olanzapine (OLZ) is one of the atypical anti-psychotics which is efficacious for schizophrenia (8). Nevertheless, OLZ-induced weight gain and metabolic disturbance and higher risk of cardiovascular diseases always were reported (9, 10).

Topiramate (TPM) has been considered to have an effect on weight and metabolism level (11, 12). A top-level evidence showed that the mid/top doses TPM had a significantly reducing effect on BMI and was related to reduced triglyceride (TG) and increased high-density lipoprotein (HDL) in adolescents with obesity (13). A systematic review and network meta-analysis of randomized controlled trials (RCT) also revealed that TPM has been considered to be the best drug in reducing weight (14). The mechanism of effect by topiramate is not well elucidated. Several assumptions were proposed in previous studies, including reduced energetic efficiency, the role of hypothalamus and neuropeptides, insulin-sensitizing effects of TPM, decreased caloric intake and hormonal involvement (15).

Moreover, TPM has a direct antioxidant property, which is reflected in neuroprotective effects (16). Previous studies have revealed that TPM could have potential protective effects on oxidative stress damage (17). It is well-known that vitamin C (VC) is a strong antioxidant. A body number of literatures have found that VC might reduce the production of oxidative stress biomarkers during exercise (18), which may increase your metabolism (19). In addition, VC also involved into iron absorption (20), which assisted in reducing body weight gain (21).

An early meta-analysis of randomized controlled trials found that TPM mitigated weight gain in antipsychotic-treated schizophrenia patients (22). In addition, a good therapeutic effect of TPM on OLZ-related weight gain and metabolism abnormalities in schizophrenia has been demonstrated in several randomized controlled trials (23–25). However, no study reported the difference in efficacy and pattern of OLZ-related weight gain and metabolism abnormalities in schizophrenia between TPM and VC. Hence, we examined the effectiveness of VC on OLZ-induced weight gain and metabolism abnormalities as well as its comparison with TPM in this study.

## 2. Methods

### 2.1. Subjects

This study was approved by the Medical Ethics Committee of Mental Health Center of Shantou University. Each patient or their guardians signed written informed consent.

Twenty-two eligible patients with schizophrenia (mean age = 27.36; females = 45.45%) who received OLZ monotherapy plus TPM treatment were recruited (OLZ + TPM group). Twenty-two additional patients with schizophrenia (mean age = 29.14; females = 50%) who received OLZ monotherapy plus vitamin C treatment (OLZ + VC group) was matched to OLZ + TPM group by a series of variables, including gender, sex, year of education, duration of illness, usage of OLZ (mg/d), BMI, WMR and proportion of positive family history of schizophrenia or other mental disorders.

The dose of olanzapine in two groups started from 5 initially to 10–20 mg/day after 1 week, if appropriate. TPM was used at an initial dose of 25 mg/day and within 1 week of therapy, gradually increased to 100–200 mg/day and maintained on the same dose. The actual dose could be increased or decreased (within range) based on the discretion of psychiatrists. VC have a same dose with TPM at the same time point.

### 2.2. Eligible criteria

All patients were recruited in this study based on the following criteria: (1) 18–60 year old; (2) having a schizophrenia diagnosis by two psychiatrists using the Chinese version of International Classification of Diseases 10th Revision, with a Kappa value greater than 0.80; (3) having a value of BMI less than 30 kg/m^2^; (4) having a total score of ≥60 on the Positive and Negative Syndrome Scale (PANSS); (5) having not taken psycho-active drugs for at least 3 months, and (6) having not participated in any clinical trial for at least 3 months.

All patients with severe cognitive dysfunction, serious physical illnesses and suicidal behaviors were excluded in this study. Subjects were also excluded if they were pregnant or lactating, or if they were planning to become pregnant.

### 2.3. Data collection and measurements

The participants were face-to face interviewed by two medical students. Basic demographic and clinical characteristics were collected by a data collection form designed for this study supplemented by a review of medical records.

The PANSS was used to assess the clinical symptoms of patients on the same day as the whole blood sampling (26). Height and Weight were recorded for five times (pre-treatment and 2-, 4-, 8-, and 12-week treatment) with a standard measure approach (27). BMI was calculated by a formula of weight (kg) divided by square of height (*m*^2^). Using a soft tape measure, waist and hip circumferences were examined with a standard WHO protocol (28). The Waist Hip Ratio (WHR) is calculated by dividing a person’s waist circumference by his/her hip circumference.

The 4 ml of venous blood were obtained from each participant on an empty stomach from 6:00 to 7:00 on the day of data collection (i.e., pre-treatment and 4- and 12-week treatment), into vacutainer tubes containing EDTA. Lipid components in blood samples, including cholesterol (CHOL), TG, HDL and low-density lipoprotein (LDL) were measured by an automatic biochemical analyzer (Hitachi Model 7,100, Japan).

### 2.4. Statistical analysis

The mean ± standard deviation (SD) for continuous variables and number plus proportions for categorical variables were employed for descriptive analyses. The Shapiro–Wilk normality tests were used to detect the normal distribution. Demographic, lifestyle behaviors variables and adverse events between the two groups were compared using the two independent samples T test, Mann–Whitney U test, Chi-squared test and Fisher exact test as appropriate.

The repeated measure analysis of variance (RMANOVA) with least—significant difference (LSD) method was used to explore patterns of patients’ BMI and lipid components over time, from pre-treatment to 12-weeks follow-up. Prior to the RMANOVA, both the normality and homogeneity tests were performed for each measurement of the independent variables. When the normality and homogeneity tests were failed, the square root transformation would be implemented. If the Mauchly’s Test of Sphericity were violated, the Greenhouse–Geisser method would be selected. The SPSS software, version 25.0 (SPSS Inc., Chicago, IL, United States) was adopted and significance was set at *p* < 0.05.

Following the previous studies (29, 30), clinically significant weight change was defined as at least 7% from the baseline. Number needed to treat (NNT) was examined as the reciprocal of the difference in rates of adverse events for OLZ + TPM group versus OLZ + VC at endpoint. A BMI equal to or over 28 is considered obese and overweight is defined as 24 ≤ BMI < 28 (31). The changes of all metabolic indexes at different time point were compared using the Mann–Whitney U test owing to the nonnormal distribution.

Latent profile analysis (LPA) was used to identify latent groups of BMI and lipid components based on the standardized Z scores. Owing to the limitation of sample size and the interpretation of classes, the “two-class” model was set. All LPA analyses were implemented using Stata version 15.1 (StataCorp, College Station, TX, United States).

## 3. Results

Overall, fourth-four patients with a diagnosis of schizophrenia who received OLZ treatment were assessed for eligibility for the study. There were not significant differences in study characteristics between the OLZ + TPM group and the OLZ + VC group (Table 1).

**Table 1:** The basic information between two groups before treatment.

Variables	OLZ + TPM (*n* = 22)		OLZ + VC (*n* = 22)		T/Z/χ^2^	*P*
	Mean	SD	Mean	SD		
Age	27.36	4.95	29.14	3.83	1.33	0.19
Year of education (years)	14.91	1.97	14.77	2.05	−0.23	0.82
Duration of Illness (years)	2.39	1.12	1.95	0.87	−1.42	0.16
Usage of Olanzapine (mg/d)	15.68	3.29	15.00	3.27	−0.68	0.50
BMI	21.96	1.70	21.81	1.81	0.27	0.79
WHR	0.87	0.03	0.86	0.03	−1.31	0.19
	*n*	%	*n*	%		
*Gender*						
Men	12	54.55	11	50.00	0.09	0.76
Women	10	45.45	11	50.00		
Family history of Psychiatry						
Yes	6	27.27	8	36.36	0.42	0.52
No	16	72.73	14	63.64		

SD, standard deviation; OLZ, olanzapine; TPM, topiramate; VC, vitamin C; BMI, body mass index; WHR, waist-to-hip ratio.

**Figure 1:**
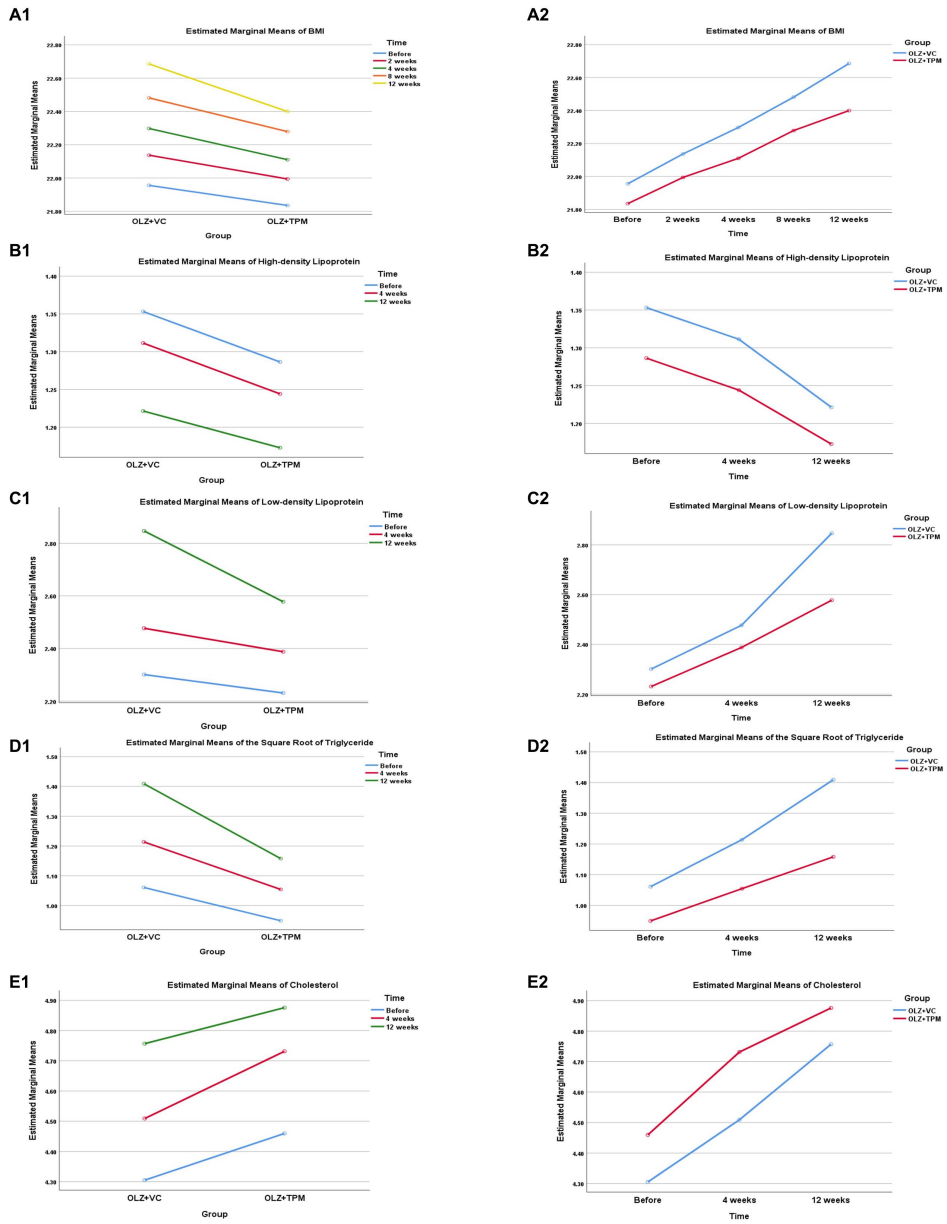
Profile plot of the patients’ metabolic indexes during 12-weeks follow-up. **(A1)** Comparison in BMI between OLZ + TPM group and OLZ + VC group at different time; **(A2)** comparison in BMI over time at different groups; **(B1)** comparison in high-density lipoprotein between OLZ + TPM group and OLZ + VC group at different time; **(B2)** comparison in high-density lipoprotein over time at different groups; **(C1)** comparison in low-density lipoprotein between OLZ + TPM group and OLZ + VC group at different time; **(C2)** comparison in low-density lipoprotein over time at different groups; **(D1)** comparison in triglyceride between OLZ + TPM group and OLZ + VC group at different time; **(D2)** comparison in triglyceride over time at different groups; **(E1)** comparison in cholesterol between OLZ + TPM group and OLZ + VC group at different time; **(E2)** comparison in cholesterol over time at different groups. OLZ, olanzapine; TPM, topiramate; VC, vitamin C; BMI, body mass index; TG, triglyceride; HDL, high-density lipoprotein; LDL, low-density lipoprotein; CHOL, cholesterol.

The RMANOVA revealed significant effect of interaction of group x time in BMI, LDL, TG and CHOL (all *p* < 0.05, Table 2), except for HDL (*p* > 0.05). All results identified that the changes of time in all metabolic indexes had a linear trend with the largest *F* values (all *p* < 0.05, Figure 1). Significant differences at different time points were found both in the OLZ + TPM group and in the OLZ + VC group (all *p* < 0.001). There was a significant difference in TG at different time points (pre-treatment: *F* = 7.89, *p =* 0.008; 4-weeks treatment: *F* = 13.19, *p =* 0.001; 12-weeks treatment: *F* = 54.48, *p <* 0.001).

**Table 2:** The changes in metabolic indexes at pre-treatment and 2-, 4-, 8-, and 12-week treatment.

Variables	Before (Mean ± SD)		Two weeks (Mean ± SD)		Four weeks (Mean ± SD)		Eight weeks (Mean ± SD)		Twelve weeks (Mean ± SD)		Time	Group	Time *Group
	OLZ + TPM	OLZ + VC	OLZ + TPM	OLZ + VC	OLZ + TPM	OLZ + VC	OLZ + TPM	OLZ + VC	OLZ + TPM	OLZ + VC	*P*	*P*	*P*
BMI	21.84 ± 1.80	21.96 ± 1.70	21.99 ± 1.81	22.14 ± 1.68	22.11 ± 1.81	22.30 ± 1.68	22.28 ± 1.83	22.48 ± 1.70	22.40 ± 1.82	22.69 ± 1.69	<0.001	0.724	0.004
BMI change	–	–	0.16 ± 0.73	0.18 ± 0.11	0.28 ± 0.09	0.34 ± 0.11	0.44 ± 0.19	0.53 ± 0.12	0.56 ± 0.19	0.73 ± 0.12	–	–	–
HDL	1.29 ± 0.30	1.35 ± 0.26	–	–	1.24 ± 0.29	1.31 ± 0.26	–	–	1.17 ± 0.28	1.22 ± 0.25	<0.001	0.462	0.057
HDL change	–	–	–	–	−0.04 ± 0.03	−0.04 ± 0.02	–	–	−0.11 ± 0.03	−0.13 ± 0.03	–	–	–
LDL	2.23 ± 0.44	2.30 ± 0.65	–	–	2.39 ± 0.47	2.47 ± 0.69	–	–	2.58 ± 0.48	2.85 ± 0.71	<0.001	0.419	<0.001
LDL change	–	–	–	–	0.16 ± 0.11	0.18 ± 0.18	–	–	0.35 ± 0.16	0.55 ± 0.18	–	–	–
TG	0.92 ± 0.25	1.14 ± 0.26	–	–	1.13 ± 0.24	1.50 ± 0.41	–	–	1.35 ± 0.21	2.00 ± 0.37	<0.001	<0.001	<0.001
TG change	–	–	–	–	0.21 ± 0.08	0.36 ± 0.31	–	–	0.43 ± 0.12	0.86 ± 0.29	–	–	–
CHOL	4.46 ± 0.44	4.31 ± 0.52	–	–	4.73 ± 0.44	4.51 ± 0.49	–	–	4.88 ± 0.45	4.76 ± 0.47	<0.001	0.247	0.001
CHOL change	–	–	–	–	0.27 ± 0.07	0.20 ± 0.08	–	–	0.42 ± 0.07	0.45 ± 0.12	–	–	–
	*n* (%)		*n* (%)		*n* (%)		*n* (%)		*n* (%)		–	–	–
Overweight	2(9.09)	2(9.09)	2(9.09)	3(13.64)	3(13.64)	3(13.64)	4(18.18)	3(13.64)	4(18.18)	3(13.64)	–	–	–

OLZ, olanzapine; TPM, topiramate; VC, vitamin C; BMI, body mass index; TG, triglyceride; HDL, high-density lipoprotein; LDL, low-density lipoprotein; CHOL, cholesterol.

There were some adverse events in both groups, such as drowsiness, dizzy, weakness, nausea, dry mouth, numbness and difficulty of concentrating. No significant difference was not observed (Table 3, all *p* > 0.05). No participant was defined as obese. The range of overweight between two groups was 9.09%–18.18%. Weight change of each participant did not exceed 7% of initial weight during the 12 weeks. BMI change (Before-12 weeks; Before-8 weeks; Before-12 weeks), HDL change (Before-12 weeks), LDL change (Before-12 weeks), TG change (Before-12 weeks) and CHOL change (Before-4 weeks) were significantly differed between two groups (all *p* < 0.05).

**Table 3:** Comparison of adverse events between two groups.

Variables	OLZ + TPM (*n* = 22)		OLZ + VC (*n* = 22)		*F*/χ^2^	*P*	NNT (95%CI)
	*n*	%	*n*	%			
Drowsiness	5	22.73	7	31.82	0.458	0.498	11(2.84; −5.85)
Dizzy	4	18.18	6	27.27	0.518	0.472	11(2.97; −6.44)
Weakness	3	13.64	5	22.73	–	0.698	11(3.15; −7.38)
Nausea	5	22.73	3	13.64	–	0.698	−11(−3.15; 7.38)
Dry mouth	4	18.18	6	27.27	0.518	0.472	11(2.97; −6.44)
Numbness	3	13.64	2	9.09	–	1.000	−22(−4.30; 7.06)
Difficulty of concentrating	3	13.64	0	0	–	0.233	−7.33(−3.57;142.09)

OLZ, olanzapine; TPM, topiramate; VC, vitamin C; NNT, number needed to treat; CI, confidence interval.

**Figure 2:**
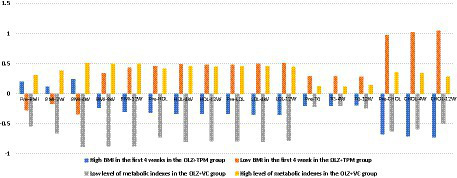
Predicted mean of BMI, triglyceride, high-density lipoprotein, low-density lipoprotein and cholesterol over time across latent profiles. OLZ, olanzapine; TPM, topiramate; VC, vitamin C; BMI, body mass index; Pre-BMI, pre-treatment BMI; BMI-2 W, 2-week treatment BMI; BMI-4W, 4-week treatment BMI; BMI-8W, 8-week treatment BMI; BMI-12W, 12-week treatment BMI; HDL, high-density lipoprotein; Pre-HDL, pre-treatment HDL; HDL-4W, 4-week treatment HDL; HDL-12W, 12-week treatment HDL; LDL, low-density lipoprotein; Pre-LDL, pre-treatment LDL; LDL-4W, 4-week treatment LDL; LDL-12W, 12-week treatment LDL; TG, triglyceride; Pre-TG, pre-treatment TG; TG-4W, 4-week treatment TG; TG-12W, 12-week treatment TG; CHOL, cholesterol; Pre-CHOL, pre-treatment CHOL; CHOL-4W, 4-week treatment CHOL; CHOL-12W, 12-week treatment CHOL.

As illustrated in Figure 2, in the OLZ + TPM group, individuals in class 1 (*N* = 13, 59.10%) labeled class 1 as “high BMI in the first 4 weeks “,” indicating high level of BMI in the first 4 weeks, but low level of other metabolic indexes. While individuals in class 2 (*N* = 9, 40.90%) had low level of BMI in the first 4 weeks, but high level of other metabolic indexes and were labeled as “low BMI in the first 4 weeks.” In the OLZ + VC group, the predicted mean of all indicators of metabolic indexes was lower than zero in class 1 (*N* = 8, 36.36%), thus it was labeled as “low level of metabolic indexes.” The predicted mean of all indicators was higher than zero in class 2 (*N* = 14, 63.64%), and it was considered as “high level of metabolic indexes.”

## 4. Discussion

To our best knowledge, this is the first study to examine the difference in efficacy and pattern of weight gain and metabolism abnormalities in OLZ-related patients with schizophrenia between TPM and VC. The findings observed that the TG levels had a significantly higher level in the OLZ + VC group than in the OLZ + TPM group over time. The trajectory of OLZ-induced weight gain and metabolism abnormalities over time also appear to be different at two groups.

Although BMI levels between two groups were not significantly different, the BMI change in the OLZ + TPM group was significantly lower than that in the OLZ + VC group from the fourth week onwards to the end. An early study revealed that OLZ-induced body weight gain could be decelerated by adjunctive TPM treatment (32), which was similar with Pandit et al. (11). The relatively small sample size and short-term follow-up could partly explain these differences between our study and other studies.

Previous studies have identified that TPM could decrease the TG level (24, 33). There are some potential mechanisms: (1) appetite suppression through hypothalamic α-amino-3-hydroxy-5-methylisoxazole-4-propionic acid (AMPA)-receptor antagonism (34, 35) (2) improvement of insulin action and glucose transport (36), and (3) inhibition of the process of *de novo* lipogenesis (37). A meta-analysis of 13 randomized controlled trials has suggested that VC could reduce serum triglyceride concentrations (38). A possible mechanism is that VC may reduce antipsychotics-induced the formation of reactive oxygen species (ROS) (39). However, when subjects start to exercise, body can generate ROS (40, 41). Thus, VC could indirectly influence metabolism. The reason for the superiority of TPM over VC may be that TPM acts directly on the metabolic processes of the body, while VC requires exercise.

The superiority of this study included long-term follow-up (12-weeks) and multiples measuring indicators. Nevertheless, there are several methodological limitations. First, the relatedly small sample size may influence the generalization of the findings in this study. Second, much more metabolic indexes, such as fasting blood glucose, etc., should be examined. Third, weight gain and metabolic abnormalities would impact the quality of life among patients with schizophrenia, but related information was not collected. Finally, relevant data on HDL, LDL, TG and CHOL in 2- and 8-week treatment were not available.

## 5. Conclusion

In conclusion, the OLZ treated patients with schizophrenia had a significantly slower rise in TG levels compared to the OLZ + VC group, when they received TPM treatment. In addition, there were a different trajectory of metabolic indexes between the two groups. A larger sample size in future studies is needed and the deeply biological mechanisms also should be further studied.

## Data availability statement

The raw data supporting the conclusions of this article will be made available by the authors, without undue reservation.

## Ethics statement

The studies involving human participants were reviewed and approved by the Medical Ethics Committee of Shantou University Mental Health Center. The patients/participants provided their written informed consent to participate in this study.

## Author contributions

W-WR, JZ, and HZ: study design. W-WR, SC, and JZ: data collection, analysis and interpretation. SC and W-WR: drafting of the manuscript. All authors contributed to the article and approved the submitted version.

## Funding

This study was funded by the science and technology projects of Shantou City (No. 221116226496181), the SUMC Scientific Research Foundation for Talents (SRFT, No.510858060), the Guangdong-Macau Youth Talent Exchange Program (No. 002–14202212).

## Conflict of interest

The authors declare that the research was conducted in the absence of any commercial or financial relationships that could be construed as a potential conflict of interest.

## Publisher’s note

All claims expressed in this article are solely those of the authors and do not necessarily represent those of their affiliated organizations, or those of the publisher, the editors and the reviewers. Any product that may be evaluated in this article, or claim that may be made by its manufacturer, is not guaranteed or endorsed by the publisher.
